# Microbial Contamination and Hygiene of Fresh Cow’s Milk Produced by Smallholders in Western Zambia

**DOI:** 10.3390/ijerph13070737

**Published:** 2016-07-21

**Authors:** Theodore J.D. Knight-Jones, M. Bernard Hang’ombe, Mwansa M. Songe, Yona Sinkala, Delia Grace

**Affiliations:** 1 Food Safety & Zoonoses Program, International Livestock Research Institute (ILRI), Lusaka 10101, Zambia; m.songe@cgiar.org; 2 Department, School of Veterinary Science, University of Zambia, Lusaka 10101, Zambia; bhangombe@unza.zm; 3 Department of Veterinary Services, Ministry of Fisheries and Livestock, Lusaka 10101, Zambia; ysinkala@gmail.com; 4 Food Safety & Zoonoses Program, International Livestock Research Institute (ILRI), Nairobi 00100, Kenya; d.grace@cgiar.org

**Keywords:** milk, safety, dairy, smallholder, Zambia

## Abstract

A field study was performed to assess safety of smallholder fresh cow’s milk around Mongu, Western Province, Zambia. This involved observation and sampling of milk along the value chain from milking to point-of-sale and storage. Samples were collected from 86 cows, from 9 farmers, selling through two dairy cooperatives, with additional samples from informal markets. Production was very low; around one litre/day/cow and 10 L/day/herd. The milk was typically transported by bicycle in high ambient temperatures without refrigeration until reaching the point-of-sale (journey times of 30–120 min), where it was sold without pasteurisation despite milk-borne zoonoses being endemic (bovine tuberculosis (bTB) and Brucellosis). Although microbiological contamination was initially low, with geometric mean total bacterial count (TBC) of 425 cfu/mL (cfu = colony forming units) upon arrival at point-of-sale, poor hygiene led to high bacterial loads later on (geometric mean TBC > 600,000 cfu/mL after two days refrigeration), with almost all samples culture positive for *Staphylococcus aureus* and *Escherichia coli*. After milking, milk was kept for 100–223 min at temperatures favouring microbial growth (median 34 °C) and sold without a microbial kill step. In this situation limited variation in observed standards of milk hygiene had no significant effect on milk end-product bacterial counts. Options for refrigerated transport are limited. Pasteurisation at the cooperative should be investigated, as this would largely remove pathogenic microbes present in the milk whether resulting from cattle infection or poor hygiene during milking and transportation. As milk is also purchased directly from producers, on-farm milk heating options should also be assessed. Smallholders may benefit from access to national markets by providing milk to large dairies, which have systems for ensuring safety. However, this requires significant investment and an increased and more consistent supply of milk; and many consumers, unable to afford milk sold through formal sectors, would not benefit.

## 1. Introduction

Like much of Africa, milk production in Western Province, Zambia, is heavily dependent upon smallholder production. Fresh milk is often sold unpasteurised to the public either directly from producers, via informal markets or through dairy farmer cooperatives. Resources are extremely limited and smallholder production is under-developed with low levels of hygiene and productivity.

Milk production cannot meet national demand in Zambia. Although consumption levels are uncertain [1,2], milk is an important source of nutrition and income in Western Zambia, with soured milk typically favoured over fresh milk [3].

Although milk-transmitted zoonoses, including bovine tuberculosis (bTB) and brucellosis, are prevalent in the Western Zambia little had been done to assess or improve milk safety and preservation [4,5]. Milk safety is crucial for both public health and farmer income, with consumers paying more for safer food [6,7]. Furthermore, improved hygiene reduces spoilage and wastage benefitting producers, traders and consumers. When untreated fresh milk is kept at ambient temperature it rapidly turns into sour milk through proliferation of lactic acid producing bacteria [8]. In Western Province, this is consumed as a mixture of curds and whey, or with the whey removed. Sour milk remains highly nutritious and the acidity inhibits many bacteria responsible for disease and spoilage. However, fresh milk is a particularly high risk perishable food, especially when consumed unpasteurised, and hence was the focus of this study [9].

Cattle are extremely important in Western Zambia for income, nutrition and status [1,3,10,11,12,13]. Farmers will often prioritise herd-size above productivity, typically only selling cattle when cash is needed and will only slaughter healthy animals for home consumption if there is a celebration. Many cattle owners live relatively impoverished lives despite often owning 50–100 cattle, each worth a few hundred US dollars. Although farmers attempt to improve their cattle through breeding and treating for parasites, few farms can be described as anything other than low-input, low-output. Most cattle are pure or crossed local breed (Barotse) with very few specialist dairy breed cattle [13].

**Figure 1:**
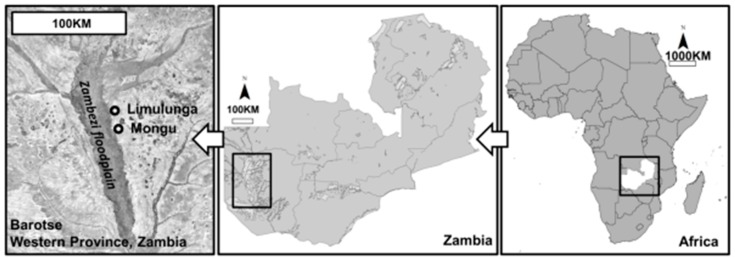
The Barotse floodplain in Western Province, Zambia. Smallholder cattle farmers were sampled that provided milk to the Limulunga (Tukongoti) and Mongu dairy cooperatives and kept their cattle in the floodplain immediately to the west of these towns.

Agriculture in the Barotse floodplain of Western Province, Zambia (Figure 1), is highly seasonal, being dominated by the annual flood of the river Zambezi and the coming of the annual rains. At times grazing is extremely lush and at other times very poor. With little forage making or supplementary feeding both body condition and milk production fluctuate over the year [10].

A value chain describes the chain of steps that a product, in this case fresh milk, passes along from production to retail and consumption, considering the various people, places and inputs involved in this process. Poor hygiene at any point from production to consumption can jeopardise final product safety, hence, analogous to Hazard Analysis Critical Control Points (HACCP), a value-chain approach is required to assess, understand and improve food safety [6].

The objective of this study was to assess levels of hygiene and bacterial contamination along smallholder milk value chains in Western Province, Zambia, focusing on fresh milk sold through smallholder dairy cooperatives near Mongu, the main town in the province. We considered where failings occur and the scope for improving milk safety.

## 2. Experimental Section

### 2.1. Planning, Data Collection and Sampling

All subjects gave their informed consent for inclusion before they participated in the study. The study was conducted in accordance with the Declaration of Helsinki, and the protocol was approved by the ILRI Ethics Committee (project identification code: ILRI-IREC2015-07).

#### 2.1.1. Scoping

An initial scoping workshop was held (February 2015) with experts in the region, mostly from provincial government agriculture, veterinary or health divisions and CGIAR food safety and nutrition researchers [14]. Subsequent meetings were arranged with participating dairy cooperatives in Western Province, Zambia (Mongu and Limulunga (Tukongoti) cooperatives).

#### 2.1.2. Selection

All producers supplying Limulunga dairy at the time of the study (March 2015) were identified and visited. In addition producers that were supplying the Mongu dairy cooperative that were identified opportunistically were also visited.

#### 2.1.3. Field Visits

**Figure 2:**
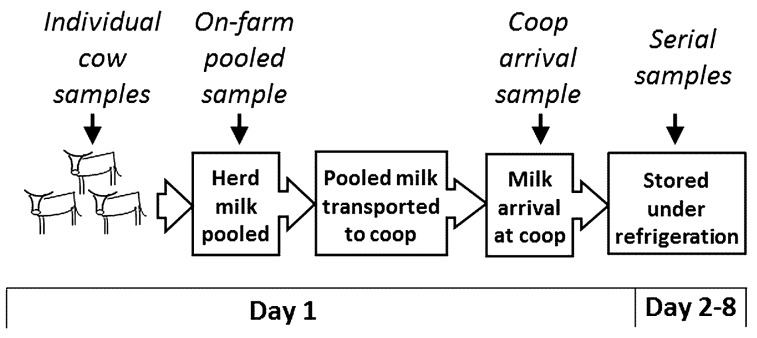
Diagram showing the flow of events during smallholder milk production and transport to the dairy cooperative where fresh milk was sold to consumers. The same batch of milk was repeatedly sampled at different points of the value chain; points of sampling are indicated.

Steps in the milk value chain and points of sampling are summarised in Figure 2.

*On farm:* Producers were visited once at milking time. Milk samples (5 mL) were collected aseptically from a single udder-quarter of all cows milked. After being milked, a cow’s milk was poured into a container containing the herd milk for that day. A milk sample was also collected from this pooled herd milk container immediately after milking. Basic data on the smallholding were collected (owner, herd-size, location). Observations were made on milking practices including measures of hygienic practice, temperature (ambient and milk) and time. Details of animal husbandry, animal health, animal births, deaths and sales were also collected (data not included). Milking and milk transportation to the dairy cooperative was done in the usual manner.

*Dairy cooperative*: After milking, the herd milk was transported to the dairy cooperative and another sample was taken (5 mL). Transportation details were recorded (time, environmental air temperature and mode of travel).

*Serial samples*: To prevent further microbial growth during storage all samples taken were kept at −20 °C from point of sampling until culturing for microbiology. However, an additional and larger (50 mL) sample of the herd milk was also collected at the cooperative. This larger sample was kept refrigerated to simulate recommended storage conditions after purchase or at the cooperative (3 °C—milk was available for sale for up to a week after milking). Smaller samples (5 mL) were then taken from this large sample (50 mL) every two days for up to eight days, with these aliquots then kept at −20 °C until microbiological testing.

*Sour milk samples*: Sour milk samples were purchased from stalls at Limulunga market (8 samples) and Mongu dairy cooperative (1 sample).

### 2.2. Microbiology and Quality Assessment

All samples were assessed for Somatic Cell Count (SCC) on farm using the California Milk Test (CMT) (Mastest, Bayer, Johannesburg, South Africa). Microbiological analyses of frozen milk samples were performed at the University of Zambia. One to two weeks after collection samples were defrosted and plated onto petri dish plates using standard methods. Total Bacterial Count (TBC) was determined using standard plate count agar (Oxoid, Basingstoke, UK). Serial dilutions were made from one mL of milk and then one mL of each dilution was plated in duplicates on Standard plate count agar and incubated at 37 °C for 18 to 24 h. The number of visible colonies was then counted. The coliform count was performed in the same way using MacConkey agar (Oxoid) and colonies exhibiting lactose fermentation were counted as coliforms. Samples were also cultured for the presence of *Staphylococcus aureus* (*S. aureus*), *Bacillus* spp., *Staphylococcus* spp., *Streptococcus* spp. and *Escherichia coli* (*E. coli*) using Blood agar (Oxoid) supplemented with 5% sheep blood cultured at 37 °C for 18 to 24 h. The bacteria were identified by colony characteristics, Gram’s staining, differential and selective media and conventional biochemical tests [15].

### 2.3. Analysis

Characteristics of cows (age, breed, milk production, number of calves and time since calving) and farms (gender of owner, herd size, location, husbandry and milking practices) were described. Hygiene, including temperature, was described for milk production and delivery to the cooperatives. Microbial presence or absence, and colony counts were described for different points of sampling along the value chain. The relationship between cattle, farm and hygiene characteristics, and the microbial quality of the milk were investigated graphically and using simple univariate statistics (chi-squared test, *t*-test, *F*-test, Pearson’s correlation coefficient and Wilcoxon rank sum test depending on the variables) with stratification as appropriate. Data were inputted using Excel (Microsoft Corp., Redmond, WA, USA) and all analysis was performed using R [16].

## 3. Results

### 3.1. Descriptive Analysis

#### 3.1.1. Herd and Cattle Characteristics

**Figure 3:**
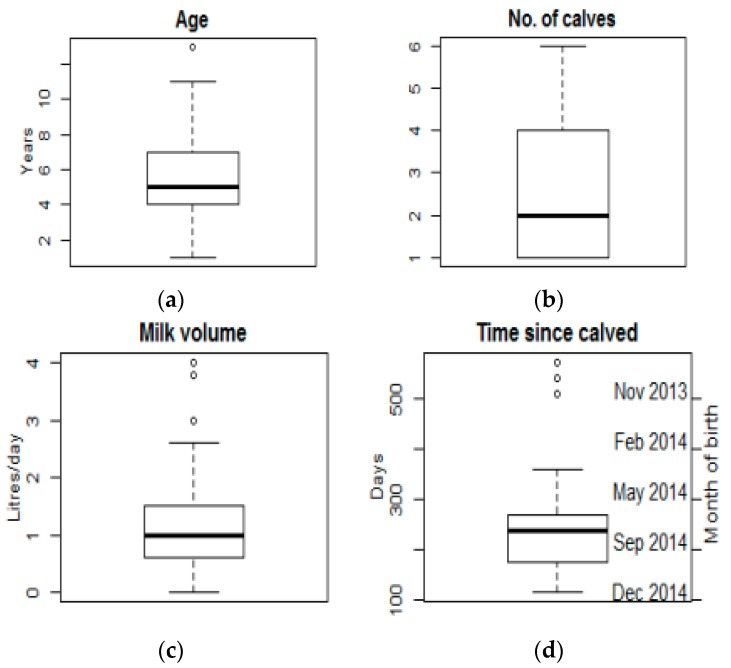
Characteristics of milking cows sampled (*n* = 86), including cow age (**a**); number of calves that cows had had in their lifetime (**b**); volume of milk collected from a cow on day of investigation (additional milk would have been drunk by calves directly from cows) (**c**); and number of days since a cow had last calved and month of last calving (**d**).

A total of 86 cows were sampled from nine herds, with 2 to 15 cows milking per herd (Figure 3). The majority of farmers (7/9, 78%) were male with two (22%) husband and wife run farms. Further details of the dairy cooperatives and cattle are included in the Supplementary Results (including Figures S1 and S2).

#### 3.1.2. Husbandry and Production Characteristics

The cattle grazed unfenced on the Zambezi floodplain at day and were kept in corrals on the floodplain at night (see photos describing the steps in the smallholder fresh milk value chain, Supplementary Figure S2), except for one farm that kept mostly dairy breeds on a fenced smallholding at night, grazing the edge of the floodplain by day. One or more herd-boys or a family member would live with the cattle all the time and the owner would visit regularly, except for the smallholder with dairy breeds who lived by the smallholding.

#### 3.1.3. Milking

Cows were milked once a day. Time of milking varied with six farmers (67%) milking between 12:30 and 16:00, the hottest time of day, two (22%) at 9:00–10:15 and the smallholding with dairy breeds milking (11%) in the cool of the morning (6:30). Milk was delivered to the cooperative immediately after milking.

Milking took 35–90 min, milking by hand into a bucket (plastic, metal or traditional wooden). Milk was then poured into a plastic (three farmers, 33%) or metal (six farmers, 67%) container that could be sealed, mostly through a muslin cloth or a sieve (8/9 farmers, 89%), which was always rinsed between cows. Unlike plastic buckets and containers, metal buckets and containers were designed for handling milk or food. Although contamination of the pooled herd milk with cattle hair was not seen, some visible dirt contamination was observed for 5/9 (56%) farms.

Milking was done by one or more herd boys. Hand washing at milking was not done on 3/7 farms (33%) (not recorded on two farms), and was subjectively scored as relatively good for one (14%) and moderate for 3/7 farms (33%). However, soap was not used and water was untreated surface water from the wetlands. This water was also used to rinse milking equipment (bucket and sieve) at the start and end of milking.

#### 3.1.4. Transport to Dairy Cooperative

Total herd milk volume at the daily milking varied uniformly from 5.5 to 15 L. Two-thirds (6/9) of farmers transported the milk to the cooperative by bicycle (one sometimes used the bus), one used motorbike or boat and one took a taxi. Journeys times varied from 30 to 120 min. Time from the start of milking to refrigeration upon arrival at the cooperative ranged from 100 to 223 min (median 113 min).

#### 3.1.5. Dairy Cooperative Practices

Limulunga cooperative had restricted farmers to provide a maximum of 10 L/day due to low demand. Conversely, Mongu cooperative could not meet customer demand for milk. Limulunga cooperative paid farmers 3 Zambian Kwacha per litre (US$0.41), Mongu cooperative paid farmers 4 Zambian Kwacha per litre (US$0.55), both cooperatives sold raw milk with a one kwacha (US$0.14) mark up. A single milk price was paid to farmers with no grading system with higher prices for better quality milk. Milk was no longer routinely tested for souring (ethanol test) or water contamination (lactometer test). Upon arrival milk was stored under refrigeration. Power cuts are frequent in the area. Milk was sold as raw fresh milk unless it went sour, in which case it was sold as sour milk. The farmer’s herd milk container was washed thoroughly at the cooperative.

#### 3.1.6. Temperature

The temperature of the pooled herd milk measured at the farm immediately after milking ranged from 30 to 38 °C. Upon arrival at the cooperative herd pooled milk temperatures ranged from 32.5 to 37 °C. Air temperature at milking ranged from 21 to 42 °C, median = 34 °C.

#### 3.1.7. Milk Microbiology and Quality

All samples were CMT negative suggesting approximate SCCs were <200,000 cells/mL [17]. Table 1 shows the milk microbiology results at all points of sampling. Although coliform counts were low, *E. coli* contamination was common as was *S. aureus* contamination, although the latter did not produce haemolytic toxin.

**Figure 4:**
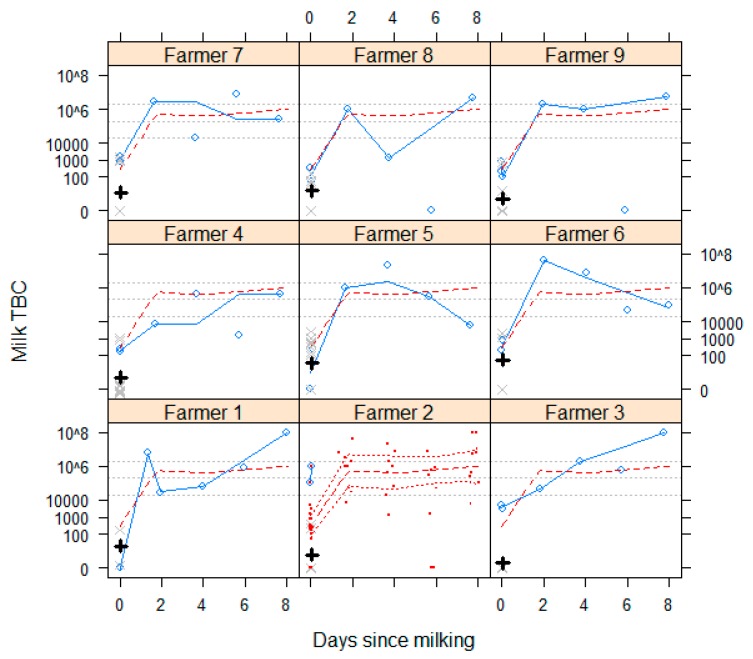
Blue circles show total bacterial counts (TBC) for pooled milk samples from each farm, taken on-farm immediately after milking and on arrival at the dairy cooperative (both days since milking = 0), followed by samples of refrigerated milk taken for up to eight days after milking. These points are joined by a blue Lowess smoothed line. Individual cow samples at milking (day 0) are shown using grey crosses with their geometric mean in black. Serial samples were not collected after arrival at the cooperative for farmer 2. A Lowess smoothed average TBC for all pooled samples from all farms is shown on all panels as a red dashed line, with upper and lower 95% confidence intervals for this Lowess all-farms’ average TBC shown on the panel for farmer 2, all TBCs for all farmers are also shown on this panel as red dots. TBC thresholds indicated by grey dotted lines show 20,000 cells/mL = EU legal limit for raw milk sold for consumption; 200,000 cells/mL = Zambia grade B upper limit and COMESA grade A upper limit; and 2,000,000 cells/mL = upper COMESA limit for milk to be processed. COMESA = Common Market for Eastern and Southern Africa.

Figure 4 shows trends in TBC for each farmer at the different sampling points after milking. There is a dramatic increase in TBC after individual cow milk was pooled on-farm in a container.

#### 3.1.8. Sour Milk

TBC was low for sour milk (Table 1), however, one sample had a TBC of 1,500,000 cfu/mL. Serial samples were taken from the Mongu cooperative sour milk sample; although having a zero TBC on the day of purchase, during refrigeration TBC rose to 4 × 10^6^ by six days, and 10^7^ by eight days after purchase.

The geometric mean TBC for sour milk that had the whey drained was 8560 cfu/mL, compared to 118 cfu/mL for undrained sour milk, however with only three drained and six undrained samples this was not significant (*p* = 0.25).

### 3.2. Univariable Analysis

Besides days since milking, no significant risk factors were found for milk microbial contamination (details reported in Supplementary Results including Figures S3 and S4).

## 4. Discussion

Although smallholder fresh milk initially had low levels of microbial contamination, absence of (1) refrigeration between milking and arrival at point of sale and (2) pasteurisation allowed rapid microbial growth resulting in high bacterial loads one to two days after milking. An absence of risk factors suggested that slight improvements in hygiene have a negligible effect on microbial quality and safety if hygiene is still limited and milk is kept at temperatures that favour rapid bacterial growth.

### 4.1. Milk Microbiology

When sampled directly from the cow the microbiological quality of the milk was very good, 95% of individual cow samples had a TBC of <1000 cfu/mL and all had a zero coliform count. TBC, which reflects overall bacterial contamination levels, increased dramatically during milking on farm, reflecting the limited hygiene of the milking process and containers used. However, on-farm milk microbiological quality was still good when compared to international standards. All but one of the on-farm pooled samples had a TBC of close to 1000 cfu/mL. Few farms in Europe or America can match this, many have TBC <5000 cfu/mL [18], and legal limits for milk collected on farm in the EU and USA are <100,000 cfu/mL [19,20].

Even after unrefrigerated transport to the cooperative outlet, TBCs were generally very low (geometric mean of 425 cfu/mL, although now one million for one farm with a high initial TBC); grade A milk in Zambia must have TBC ≤50,000 cfu/mL [21]. However, once this initial lag phase had passed limited hygiene and high transport temperature [18] resulted in high bacterial counts, with geometric mean TBC of >600,000 cfu/mL 1–2 days after arrival at the cooperative, despite refrigeration after arrival at the cooperative (1–2 h after milking). From the cooperative milk was sold as unpasteurised raw milk. In the EU a TBC <20,000 cfu/mL is required for raw milk sold for consumption [19,20].

TBCs were generally lower than other studies of smallholder microbial milk quality in Zambia which reported mean TBCs of <10,000–100,000 cfu/mL [21,22]. Studies of smallholder milk elsewhere in Sub-Saharan Africa find even higher bacterial loads; studies in Rwanda, Kenya, Ethiopia and Cameroon reported mean TBCs of one million to 100 million cfu/mL for raw milk arriving at milk collection centres [23,24,25,26]. However, higher contamination levels were reported in a 1996 study of commercial farms around Lusaka, Zambia (log10(Standard Plate Counts) = 7.6–9 cfu/mL) [27]. These differences partly correlate with differences in milk yields, with higher TBCs found in cows with greater yields associated with increased udder infection [27].

In our study samples were frozen for one to two weeks before culturing as samples could not be cultured in the field. However, less than three weeks freezing is thought to have a negligible effect on TBC, although it may cause a slight reduction in coliform and even gram positive counts [28,29,30].

Poor hygiene is reflected by the high proportion of samples contaminated with *Staph. aureus* and *E. coli*, particularly after storage, suggesting poor handling and faecal contamination respectively [31]. That said, coliform counts remained low, with a geometric mean close to zero until 8 days after milking, when it rose to 20; English legislation requires coliform counts of <100 cfu/mL for milk to be drunk raw and the EU requires 0–5 cfu/mL for pasteurised milk [19,32,33]. This is not to say that consuming the evaluated smallholder raw milk is recommended, as dangerous milk-borne zoonoses not assessed in this study, including *Brucella abortus* and *Mycobacterium bovis* which would be killed by pasteurisation [34], are carried by the local cattle population [4,5,35]. Other milk-borne pathogens not assessed included *Toxoplasma gondii* and several diarrhoea causing microbes, such as *Salmonella* spp. and *Campylobacter* spp. which account for a large fraction of the burden of foodborne disease, particularly in Africa [35,36].

Udder health appeared to be good, with no positive CMT tests and with low levels of isolation of common mastitis pathogens. A quarter of cow samples had *S. aureus* growth, however, this could reflect poor teat hygiene rather than udder infection, particularly as calves drank milk directly from cows.

Sour milk microbiological quality was similar if not better than fresh milk at point of purchase, except for *Bacillus* spp. This does not reflect better hygiene; the sour milk sold would have been several days old at point of sale, often transported by foot from herds deep in the flood plain, and it is made from pooling milk from several herds, which increases contamination risk [37]. However, the acidic conditions of sour milk inhibit many pathogenic bacteria, although with imperfect efficacy [9,38].

### 4.2. Observed Hygienic Practice

Most critical to milk safety was the lack of pasteurisation or boiling, which would kill off almost all microbial pathogens present [34]. The long time that milk was kept at a high temperature, on farm and during transport was also important (approximately 35 °C for ≥45 min). Farmers attempted to minimise contamination during milking, however, they lacked the resources to do this effectively.

Overall the findings are not surprising. Milk typically has little to no contamination when sampled directly from healthy cows. Poor hygiene results in bacterial contamination, with initial inhibition of bacterial growth for a few hours resulting from the inherent antibacterial properties of milk, followed by a lag phase before rapid microbial growth, which is inhibited by refrigeration [18,35,39].

The longitudinal sampling approach adopted in this study allowed observation of changes in bacterial contamination along the milk value chain. Sampling at a single point may have led to different conclusions about the levels of milk contamination, i.e., low contamination on farm and at arrival at the dairy cooperative, variable contamination one day later or consistently high levels of bacterial contamination after two or more days’ storage.

### 4.3. Risk Factors

No significant risk factors for final milk microbial quality were detected. Even when better hygiene is practiced at one point of the value chain, limited hygiene at other steps allowed microbial contamination with subsequent bacterial multiplication, resulting in similar microbial quality of the end product regardless of variation in upstream hygienic practices. Ensuring end-product microbial safety requires (1) all aspects of production to be hygienic or (2) a final kill-step to remove upstream contamination, with subsequent hygienic handling and refrigeration.

Limitations of the study also contributed to the absence of detected risk factors. Production methods were similar for all farms resulting in limited variation in risk factors; the small number of farms sampled led to limited power for detecting herd-level effects; and the microbial culture assays used have inherent variability, leading to reduced power to detect differences between groups.

### 4.4. Options for Improved Milk Safety

Much is already known about the requirements for safe milk production and future work should look at the effect and feasibility of interventions to improve milk quality.

#### 4.4.1. Funding and Pricing

Funding is required to improve safety. This requires a higher milk price to be paid by customers or outside funding, recognising the reduced societal burden of disease arising from safer milk. Although consumers may pay more for safer milk the low level of production limits the revenue that can be raised through increased milk price unless output is scaled up. Furthermore, those that cannot afford more expensive but safer milk may be forced to buy cheaper less safe milk elsewhere.

Currently all farmers receive a single milk price, regardless of milk quality. Paying producers more for safer and higher quality milk would create an incentive for producer investment in milk quality.

#### 4.4.2. Refrigeration

High costs and inadequate infrastructure (roads, electricity) make refrigeration at point of production or refrigerated transport seemingly unfeasible. Milk temperatures during transport (32–37 °C), were just below the temperature at which most milk microbial pathogens cannot grow (generally 37–48 °C, 57 °C for *Salmonella* spp.) [34]. Transporting milk at deliberately raised temperatures to prevent microbial growth would be a novel and speculative approach that could be investigated.

#### 4.4.3. Pasteurisation

Pasteurisation at the dairy cooperative or a subsequent processing centre is likely to be effective and would be easier to implement compared to interventions applied to all farmers. However, some of the milk is sold directly from the farm or consumed by the farmer’s family.

A problem with on-farm pasteurisation is that subsequent microbial proliferation will occur during unrefrigerated transport to the cooperative. Furthermore, it is harder to ensure pasteurisation is done correctly by many producers compared to one central processor. However, options for pasteurisation at the cooperative need to consider the limited infrastructure (reliable electricity, technical support, etc.), scales of production and funding. Solar heating may be worth considering. As well as reducing carbon emissions, solar technologies are particularly suitable to the floodplain where there is a lack of wood, however, during the wet season, frequent clouds make solar heating less reliable and a back-up alternative would be required.

It must be noted that poor hygiene cannot be entirely mitigated by pasteurisation particularly if milk is heavily contaminated or if handling after pasteurisation is unhygienic. Also pasteurisation does not inactivate toxins produced by some strains of *S. aureus* [40], commonly found in poorly handled smallholder milk [41]. As milk does not display a visible change when pasteurised, there is a risk that pasteurisation will not be done adequately. Boiling requires more energy but is easily observed and may therefore, be more reliable [42] when temperature cannot be easily monitored [43].

#### 4.4.4. Awareness

Although participants seemed aware that consuming raw milk could cause illness, other studies have found a poor understanding of milk-borne disease in rural communities in Zambia [44,45]. Raising awareness of the importance of heating raw milk before consumption may be beneficial [46] and may increase willingness to pay for pasteurised milk [7].

Although less effective than pasteurisation, fermenting milk reduces microbial contamination compared to raw milk [38]. If fresh milk is to be consumed raw, promoting rapid consumption after milking would also be sensible to reduce the time available for microbial multiplication.

A better understanding of consumer preferences and willingness to pay is needed, considering raw, pasteurised, boiled and soured milk. Fundamental issues surrounding scales and seasonality of production in relation to improved milk safety, and how to help those least able to afford safe food are discussed in the Supplementary Discussion.

## 5. Conclusions

Smallholders in Western Province produce milk of good initial quality but in very small quantities (a litre per cow/day and about 10 L per herd/day). However, levels of hygiene are low with no refrigeration of milk until it arrives at the point of sale, where it is sold without pasteurisation. Zoonoses, such as bTB and Brucellosis, are present in the local cattle population, and levels of bacterial contamination of milk are high. The result is a high-risk product with rapid spoilage. In this under-developed setting, options for improving milk safety are limited. However, sustainable methods of milk pasteurisation should be investigated as a microbial kill-step is needed to mitigate upstream contamination.

Significant investment in safety is easier to justify when the milk supply is sufficiently large and reliable, with large, reliable markets. Opening up markets through development of the dairy sector could increase milk safety and farmer income, however, a large proportion of consumers are unable to afford milk sold in formal markets and simple, affordable methods of small-scale pasteurisation should also be investigated.

## Figures and Tables

**Table 1:** Table showing microbiological test results for milk sampled at different points along the smallholder fresh milk value chain in Western Zambia.

Outcome	Point of Sampling								
	Cow		On-Farm Pooled Herd Sample	Coop Arrival	1–2 Days	4 Days	6 Days	8 Days	Sour Milk on Day of Purchase
	All Cow Samples	Within Herd Prevalence Median (min–max)							
Bacterial growth	26/86 (30%)	29% (0%–57%)	8/9 (89%)	8/9 (89%)	9/9 (100%)	8/8 (100%)	6/6 (100%)	8/8 (100%)	8/9 (89%)
*Staphylococcus aureus* *	19/86 (22%)	22% (0%–50%)	6/9 (67%)	7/9 (78%)	8/9 (89%)	7/8 (88%)	6/6 (100%)	8/8 (100%)	2/9 (22%)
*Escherichia. coli*	11/86 (13%)	0% (0%–50%)	6/9 (67%)	8/9 (89%)	9/9 (100%)	8/8 (100%)	6/6 (100%)	8/8 (100%)	4/9 (44%)
*Bacillus* spp.	7/86 (8%)	7% (0%–25%)	0/9 (0%)	0/9 (0%)	3/9 (33%)	3/8 (38%)	1/6 (17%)	6/8 (75%)	5/9 (56%)
*Staphylococcus* spp.	11/86 (13%)	11% (0%–50%)	2/9 (22%)	6/9 (67%)	8/9 (89%)	8/8 (100%)	6/6 (100%)	7/8 (88%)	2/9 (22%)
*Streptococcus* spp.	2/86 (2%)	0% (0%–15%)	0/9 (0%)	0/9 (0%)	1/9 (11%)	0/8 (0%)	0/6 (100%)	0/8 (0%)	0/9 (0%)
TBC > 1000	4/86 (5%)	0% (0%–50%)	3/9 (33%)	2/9 (22%)	9/9 (100%)	8/8 (100%)	6/6 (100%)	8/8 (100%)	3/9 (33%)
*Geometric mean*	*6*	*7 (1*–*39)*	*269*	*425*	*630,000*	*345,000*	*213,000*	*1,416,000*	*491*
Coliform count > 0	0/86	All negative	0/9 (0%)	0/9 (0%)	1/9 (11%)	1/8 (12%)	0/6 (0%)	3/8 (37%)	0/9 (0%)
*Geometric mean*	*-*	*-*	*-*	*-*	*2*	*3*	*-*	*20*	*-*

* All *S. aureus* isolates were non haemolytic-toxin producing.

## Supplementary Materials

The following are available online at www.mdpi.com/1660-4601/13/7/737/s1, Figure S1: Number of cows in milk for herds sampled from the two dairy cooperatives; Figure S2: Arrows show the order of steps in the Western province smallholder fresh milk value chain; Figure S3: Plots showing contamination (percent positive or geometric mean cfu/mL) according to different microbiological outcomes for pooled herd samples collected on farm, in the dairy coop and subsequent periodic samples of refrigerated milk after arrival at the dairy cooperative for each farmer; Figure S4: Plots showing contamination (percent positive or geometric mean cfu/mL) according to different microbiological outcomes for pooled herd samples collected on farm, in the dairy coop and subsequent periodic samples of refrigerated milk after arrival at the dairy cooperative for each farmer.
