# Supplementary material for: Microbial Contamination and Hygiene of Fresh Cow’s Milk Produced by Smallholders in Western Zambia
Source: Int J Environ Res Public Health. 2016 Jul 21;13(7):737. doi: 10.3390/ijerph13070737 (PMC4962278; doi:10.3390/ijerph13070737)
Supplement: Supplementary file 1 [file ijerph-13-00737-s001.pdf]

Supplementary Materials: Microbial Contamination and Hygiene of Fresh Cow's Milk Produced by Smallholders in Western Zambia

Theodore J.D. Knight-Jones, M. Bernard Hang'ombe, Mwansa M. Songe, Yona Sinkala and Delia Grace

1. Supplementary Results

Further dairy and animal characteristics (Figure S1) and description of the steps in the Western province smallholder fresh milk value chain (Figure S2).

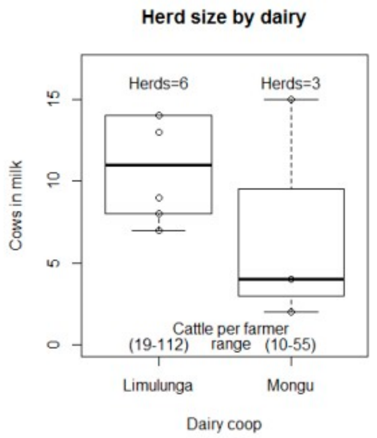

Figure S1. Number of cows in milk for herds sampled from the two dairy cooperatives. Total cattle per farm (herd-size) is also shown.

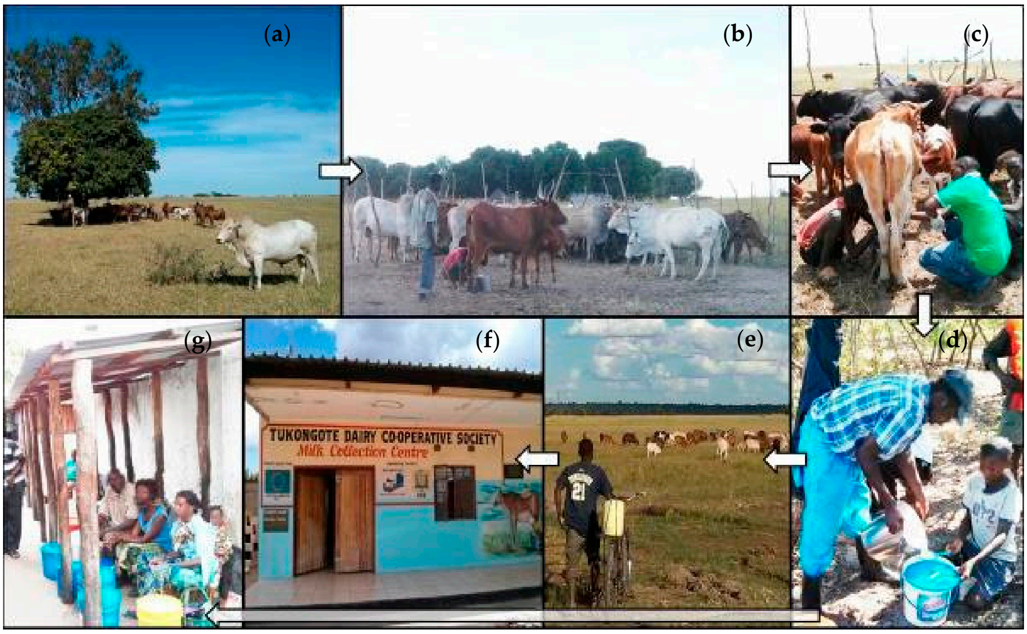

Figure S2. Arrows show the order of steps in the Western province smallholder fresh milk value chain. Milk is purchased by consumers either directly from the farmer, from the dairy cooperatives or from the informal market. Photos: Bull on the floodplain (**a**); cattle corralled and being milked (**b**), milk sample being taken at milking (**c**); pouring milk through a sieve from the milking bucket into the collection container (**d**); transportation of milk to the dairy cooperative by bicycle (**e**); Limulunga dairy cooperative (**f**), milk vendors at Limulunga market (**g**).

From sampled cattle 44 (51%) were Barotse local breed, 26 (30%) were Boran crosses, 9 (10%) were Brahman and 13 (15%) were dairy breeds (Jersey or Friesian). Suckling calves were always kept with the milking cow (82, 95%) unless sold, dead or old enough to self-wean.

1.1. Univariable Analysis

1.1.1. Cow Samples

An association between whether or not bacterial growth occurred in cow milk samples and cow-level predictors was not detected for any variables; cow age (Chi-squared p = 0.3), breed (Chi-squared p = 0.96), number of calves (Chi-squared p = 0.8), milk volume (F-test p = 0.1) or days since last calving (F-test p = 0.1).

1.1.2. Pooled Herd Samples

An association was not detected between pooled herd sample TBC and temperature of milk immediately after milking (*rho* = 0.07, p = 0.6) or at arrival at the coop (*rho* = 0.3, p = 0.09), nor was TBC associated with air temperature at milking (*rho* = −0.04, p = 0.8). Results remained non-significant if Log_e(TBC + 1) was assessed as the outcome to normalise the data. Time of milking and delivery of milk had no effect on milk microbiology (both p = 0.8).

Duration of milking had no effect on TBC (*rho* = −0.1, p = 0.5). Dirt in milk was not associated with TBC (t-test p = 0.1) or any other bacterial measures (Figure S3), 97% versus 90% had bacterial growth upon culturing for those with and without visible dirt in pooled milk after milking. Filtering the milk did not affect the proportion of samples with bacterial growth (Chi-squared p = 0.6).

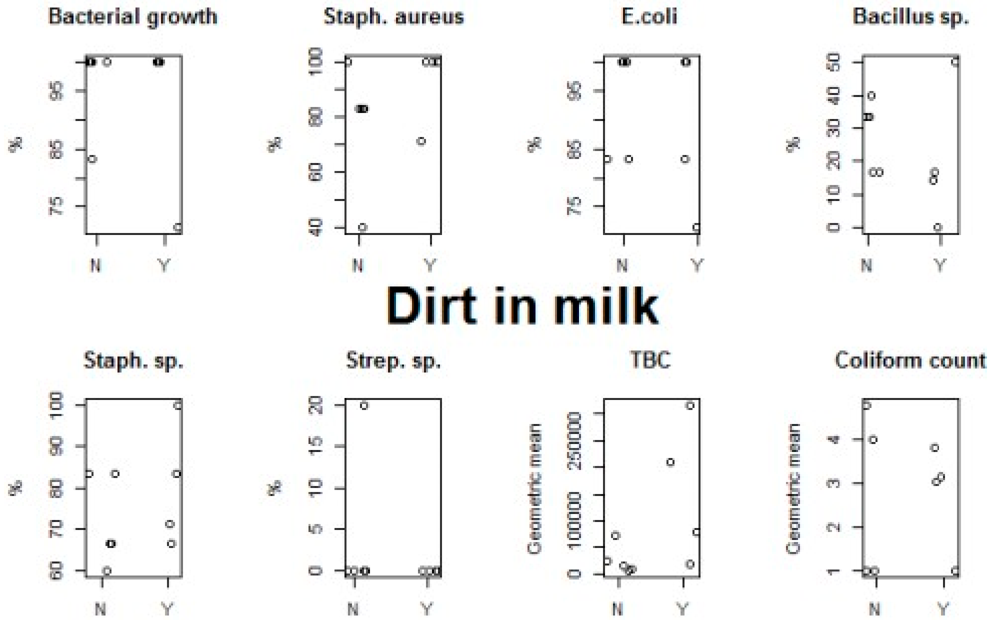

Figure S3. Plots showing contamination (percent positive or geometric mean cfu/mL) according to different microbiological outcomes for pooled herd samples collected on farm, in the dairy coop and subsequent periodic samples of refrigerated milk after arrival at the dairy cooperative for each farmer. Farms are categorized based on whether dirt was seen in farm pooled milk (x-axis).

Similarly, the use of a plastic versus a metal container for transporting the herd milk was not associated with a change in TBC (t-test p = 0.4), nor was hand hygiene (t-test p = 0.7), nor the gender of the farm owner (Wilcoxon rank sum p = 0.2), or herd size (Pearson's *rho* = 0.3, p = 0.1). Method of transport of milk had no effect on pooled milk TBC (F-test p = 0.9), nor did time taken to transport milk to the coop (*rho* = 0.04, p = 0.8) (Figure S4).

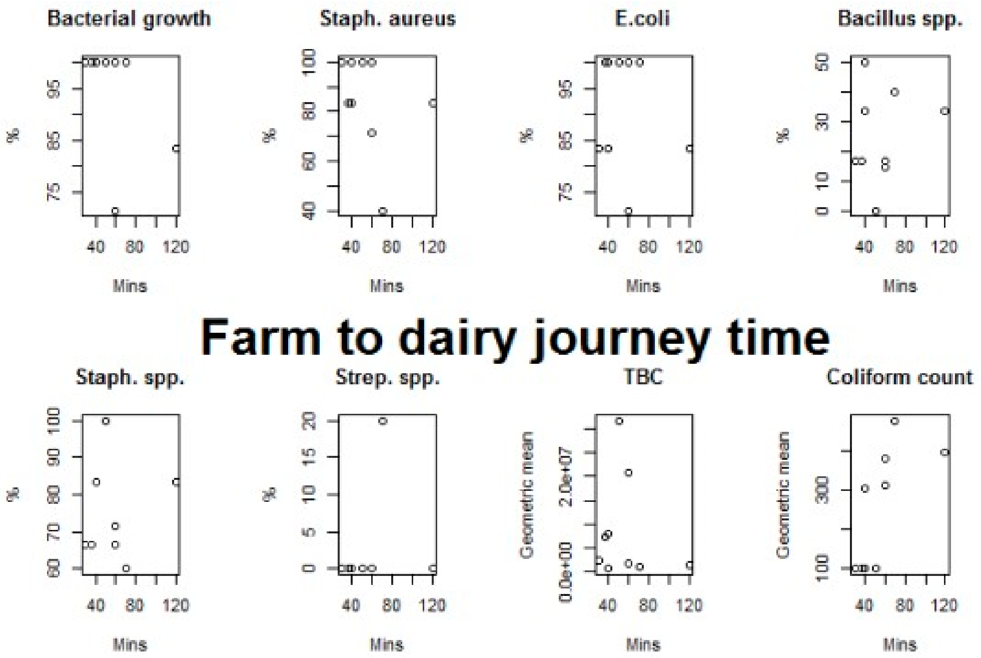

Figure S4. Plots showing contamination (percent positive or geometric mean cfu/mL) according to different microbiological outcomes for pooled herd samples collected on farm, in the dairy coop and subsequent periodic samples of refrigerated milk after arrival at the dairy cooperative for each farmer. Journey time for milk from the place of milking to the dairy coop is shown on the x-axis.

2. Supplementary Discussion

2.1. Catch-22: Which Comes First, Scaling Up Production or Market Access, Infra-Structure and Quality?

Accessing formal markets would not only increase producer revenues, but should potentiate better safety and quality through improved processing methods, incentives for milk quality and regulation. Although there is a large national demand for milk, large dairies processing fresh milk did not collect milk from Western Province (*since completion of the study a large dairy has established a processing plant in Mongu producing sour milk*).

Smallholders do provide milk to large dairies elsewhere in Zambia. However, for large dairies, new milk collection centres cannot be built too far from existing ones, or else the cost of milk collection becomes too high. Thus dairy development spreads out from existing supply chains, along good transport routes. So although the large dairies sell fresh milk in Western Province, none of this milk is produced in the region, despite there being half a million cattle in Western Province, with carrying capacity for many more [1].

The problem is more complex. In order for large scale dairies to collect milk, a large, reliable supply of milk is required, otherwise the cost of installing a processing centre cannot be justified (*this is an issue for the newly installed processing plant*). Although investments could be made to increase milk production, farmers would need to have a market for this milk, which will not exist until large dairies are able to collect their milk, which again requires increased production with production efficient enough to compete on the national scale. Investment is required to overcome this *status quo*.

Investing to improve market access first will create incentives to increase production and quality, which would then require additional support if smallholders are to fully benefit. *An increased and more reliable milk supply is indeed needed if the newly established Mongu milk processing plant is to be viable in the medium term, some farmers will be more able to respond to this demand than others.*

2.2. Challenges and Options for Increased Production

Increasing production is in fact not simple. A number of improvements would be required, including better year-round nutrition and forage-making, as well as improved breeds, necessitating

better health-care, nutrition and husbandry, without which improved breeds are likely to die. The most efficient way of introducing improved breeds is through artificial insemination which requires technical support, healthy well-conditioned cattle and infrastructure that has previously failed in the region.

Rapidly moving from a low-input, low-output system to a high-input, high-output system, which characterises commercial dairy production, would be over-ambitious with a high-risk of failure. Small steps should be taken improving nutrition, husbandry and health first. This will itself increase productivity and allow improved but suitable breeds to be introduced that are better able to utilise these improved inputs to increase productivity, yet are still able to thrive in that setting.

2.3. Challenges of Seasonal Migration and Production

The seasonal rains, grazing and migration of livestock in the Barotse floodplain creates yet another problem for milk value-chains. After calving (June–November, Figure 3d) milk production increases and peaks with improvements in grazing, brought by the rains in October-December [2–6]. However, this occurs when cattle are in the floodplain and further from the cooperatives and consumers, meaning longer unrefrigerated journeys for the milk, with many producers unable to send fresh milk to the cooperatives. Milk production declines with further time since calving and deteriorating grazing as the rains dry up (typically after February–March), and the floodwaters advance (peaking around April) forcing cattle to concentrate on the high ground where the towns and cooperatives are located. From May–July the retreating floodwaters leave behind improved grazing and cattle move back into the floodplain and start to calve, with the Zambezi river at its smallest, and the area of floodplain grazing at its maximum, around November [4]. Thus not only is milk supply highly variable but getting the milk to consumers, processors and vendors is most difficult when milk production is high and vice versa. This favours the sale of soured milk which is very popular in the region, as opposed to fresh milk which is highly perishable. Furthermore, if milk intended for sale as raw fresh milk turns sour, it is typically sold as sour milk. Pasteurisation affects the souring process and the impact of this would need to be considered [7].

2.4. Helping Those Least Able to Afford Safe Food

Improving quality and safety costs money. Customers do pay more for trusted, pasteurised milk; 7–8 kwacha (US$0.96–1.1)/litre in the Mongu supermarket, versus 4–5 kwacha (US$0.55–0.7) for raw milk from the coop at the time of the study. Furthermore, reducing microbial contamination not only improves safety but reduces spoilage creating a longer-lasting product.

However, many consumers would not be able to afford more expensive milk and would end up buying cheaper raw milk from other sources [8]. In fact many already purchase milk from informal markets paying 2–3 kwacha (US$0.3–0.4) but for only a quarter of a litre. The sustainability of a subsidised system as a public health measure requires consideration.

Of course, subsidising safety though a centralised processor would impact on the livelihoods of informal milk traders selling less safe, unprocessed milk. Furthermore, food safety in formal markets in developing countries is not necessarily better than in informal markets. As informal markets provide for the poor majority, who lack resources to fund improved food safety themselves, there is a strong case for external funding for improved safety in this sector, building on existing informal value chains [9,10]. Levels of under-nutrition in Zambia are amongst the highest in the world [11]. Increasing the supply of safe but affordable milk could help improve this situation, particularly for children.

References

1. Eroarome, M.A. Country Pasture/Forage Resource Profiles—Zambia II. FAO Report. 2006. Available online: http://www.fao.org/ag/agp/agpc/doc/counprof/zambia/zambia2.htm (accessed on 1 March 2016).
2. Baars, R.; De Jong, R.; Zwart, D. Costs and returns of the crop-cattle system in the Western Province of Zambia. *Rev. Elev. Med. Vet. Pays Trop.* **1996**, *49*, 243–251.

3. Baars, R.; Jeanes, K. The grazing capacity of natural grasslands in the Western. *Trop. Grasslands* **1997**, *31*, 561–568.
4. Baars, R.; Ottens, J. Grazing behaviour and diet selection of Barotse cattle on a communally grazed floodplain in west Zambia. *Afr. J. Range Forage Sci.* **2001**, *18*, 5–12.
5. Van Klink, E. Aspects of Productivity of Traditionally Managed Barotse Cattle in the Western Province of Zambia. Ph.D. Thesis, Wageningen University, Wageningen, The Netherlands, 2 November 1994.
6. Van Klink, E.; Corten, J.; Kalokoni, D. Herd monitoring in traditional cattle husbandry as a tool for productivity research and livestock development. *Trop. Anim. Health Prod.* **1996**, *28*, 273–279.
7. O'Connor, C.B.; Tripathi, B.R. *Milk Processing Techniques—Sour Milk, Rural Dairy Processing Training Series*; International Livestock Centre for Africa: Nairobi, Kenya, 1992.
8. Grace, D. Zoonoses–Infections affecting humans and animals. In *Zoonoses of Poverty: Measuring and Managing the Multiple Burdens of Zoonoses and Poverty*; Sing, A., Ed.; Springer Science: Berlin, Germany, 2015.
9. Roesel, K.; Grace, D. *Food Safety and Informal Markets: Animal Products in Sub-Saharan Africa*; Routledge: London, UK, 2014.
10. Grace, D. Food Safety in Low and Middle Income Countries. *Int. J. Environ. Res. Public Health* **2015**, *12*, 10490–10507.
11. UNICEF, Zambia—Nutrition, 2015. Available online: http://www.unicef.org/zambia/5109_8461.html (accessed on 1 March 2016).

© 2016 by the authors. Submitted for possible open access publication under the terms and conditions of the Creative Commons Attribution (CC-BY) license (http://creativecommons.org/licenses/by/4.0/).
